# Characterization of the Microstructure Evolution in IF-Steel and AA6016 during Plane-Strain Tension and Simple Shear

**DOI:** 10.3390/ma8010285

**Published:** 2015-01-15

**Authors:** Gregory Gerstein, Benjamin Klusemann, Swantje Bargmann, Mirko Schaper

**Affiliations:** 1 Institute of Material Science, Leibniz Universität Hannover, Garbsen 30823, Germany; E-Mail: gerstein@iw.uni-hannover.de; 2 Institute of Continuum Mechanics and Material Mechanics, Hamburg University of Technology, Hamburg 21073, Germany; E-Mail: benjamin.klusemann@tuhh.de; 3 Institute of Materials Research, Helmholtz-Zentrum Geesthacht, Geesthacht 21502, Germany; 4 Lehrstuhl für Werkstoffkunde, Universität Paderborn, Paderborn 33098, Germany; E-Mail: schaper@lwk.uni-paderborn.de

**Keywords:** microanalysis, mechanical characterization, sheet forming, fracture, electron microscopy

## Abstract

In the current work, the evolutions of grain and dislocation microstructures are investigated on the basis of plane strain tension and simple shear tests for an interstitial free steel (DC06) and a 6000 series aluminum alloy (AA6016-T4). Both materials are commonly-used materials in the automobile industry. The focus of this contribution is on the characterization and comparison of the microstructure formation in DC06 and AA6016-T4. Our observations shed light on the active mechanisms at the micro scale governing the macroscopic response. This knowledge is of great importance to understand the physical deformation mechanisms, allowing the control and design of new, tailor-made materials with the desired material behavior.

## 1. Introduction

In today’s industrial manufacturing of sheet metal products, highly-specialized alloys are used to improve the properties of the final product and/or to reduce the total costs of production. The use of high strength steels and aluminum alloys is desired, because lower weight is achieved at comparable structural stiffness, e.g., in automotive body parts. In order to fully exploit these materials, deep drawing as a manufacturing process is simulated using continuum mechanical models and the finite element method. The material behavior is described by the constitutive formulation of the stress. Material models commonly used (e.g., based on the classical works of von Mises [1] and Hill [2] or more recent works by Barlat *et al*. [3]) describe the material behavior at the macroscopic scale in a phenomenological manner. Central to these models is that their physical motivation originates in the interpretation of the stress-strain data of mechanical tests, such as uniaxial tension or simple shear tests.

There exist several experimental investigations on the strain path-dependent behavior of interstitial free steel (IF steel); see, e.g., [4,5,6,7,8,9,10,11,12]. Ghosh and Backofen [6], for example, report an increase in yield stress for certain sequences of biaxial stretching. Later, these transitions in the stress-strain curves are related to the formation of persistent dislocation structures and their interactions with dislocations associated with the subsequent loading direction; see, e.g., [5,9,10]. Fernandes and Schmitt [5] show that under load path changes (uniaxial to biaxial tension) in low carbon steel, the dislocation structures are comparable to the ones under monotonic loading. Their work also indicates that the dislocation structure saturates for large strain values, as the morphology does not evolve with further applied strain. Wilson and Bate [11] find that the formed dislocation structure mainly determines the hardening behavior under load path changes; other properties, such as grain size, grain morphology and texture, are only of minor importance. The recent work of Yin *et al*. [12] investigates the material behavior of DC06 under shear conditions and provides information about the comparability of different shear test configurations and the homogeneity of the shear zone. van Riel [13] studied the mechanical behavior of DC06, dual-phase steel DP600, high strength steel H340LAD and aluminum alloy AA5182 under strain path changes. In addition to the observed Bauschinger effect (*i.e*., a significant decrease of yield stress after reverse loading [14]), strong cross-hardening is observed for DC06 under orthogonal load path changes.

Aluminum alloy AA6016-T4 is investigated by Zillmann *et al*. [15]: the stress-strain behavior in uniaxial and biaxial compression tests is analyzed, and a biaxial tension-compression symmetry is observed. In AA6016, the Bauschinger effect is documented by shear tests with load reversal after different amounts of forward prestrain, *cf*. [16]. Hasegawa *et al*. [17] correlated the Bauschinger effect and the decrease in hardening rate after such a change with the evolution of the dislocation microstructure in pure aluminum. In particular, the decrease in hardening rate is linked to the partial dissolution of cells after load reversal.

Mathematical modeling approaches associated with such micro-macro interactions differ by the scale at which the mathematical constructs and their emerging relations are formulated. The microscale-based mechanistic approaches use mathematical entities directly linked to corresponding microstructural phenomena. In general, plastic yielding is related to the formation and interaction of dislocations in single-phase materials with few precipitates. As a consequence, a detailed model requires the modeling of the dislocation interaction on a scale of a few microns (e.g., [18,19,20,21,22,23,24,25,26,27]). However, in forming simulations, this is computationally too expensive. In phenomenological models, macroscopic work hardening behavior is expressed in terms of a function of equivalent plastic strain. The latter can be interpreted as a measure for the intensity of interactions of dislocations. Phenomenological models of this class yield acceptable results simulating sheet metal forming processes; see, e.g., Roll *et al*. [28] for single-phase materials, such as HC260LAD, Barthel *et al*. [29] for LH800 or Behrouzi *et al*. [30] for DC06. A recent model by Rabahallah *et al*. [31] includes crystallographic information on grain orientations in the computation of coefficients describing the initial point of yielding. However, most phenomenological models do not include crystallographic information in the description of plastic anisotropy resulting from texture.

Most of these models are not suited to relate the stress-strain curve for a given deformation path to the results gained by optical analysis of the grain and dislocation microstructure. Important contributions to the combination of knowledge gained from the investigations by transmission-electron microscopy (TEM) analysis of single and polycrystalline metals subjected to mechanical loading combined with a phenomenological modeling approach are made by the group of Teodosiu (e.g., Hu [32], Teodosiu and Hu [33], Bouvier *et al*. [34]). These works result in a phenomenological model for the transient behavior of polycrystalline sheet metals after changes in strain path.

Regardless of the chosen modeling approach, material modeling motivated by analysis of the microstructure requires the knowledge of active mechanisms at given levels of deformation and strain mode for different classes of materials. The goal of this investigation is to provide further insight into the microstructural evolution, *i.e*., the dislocation patterns, in DC06 and AA6016-T4 for the future development of micromechanical motivated models for application in forming processes.

## 2. Materials and Material Testing

### 2.1. Interstitial Free Steel DC06 and Aluminum Alloy AA6016

Interstitial free steel DC06 (sheet thickness 1 mm) delivered by ThyssenKrupp Steel Europe AG is investigated. Table 1 states the chemical composition according to DIN EN 10130:2006 [35]. After cold rolling, the material was annealed and subjected to a final skin pass by the manufacturer. The average Young’s modulus *E* over the three directions 0°, 45° and 90° with respect to the rolling direction was determined as 181 GPa with Poisson’s ratio *ν* = 0.3 [4].

**Table 1:** Chemical composition (mass %) of DC06 according to DIN EN 10130:2006 [35], determined by the manufacturer (ThyssenKrupp Steel Europe) by means of an analysis of one single specimen.

Alloying Component	C	Si	Mn	P	S	Al	N	Ti	Fe
weight percentage	0.003	0.018	0.137	0.013	0.010	0.035	0.0027	0.079	balance

The second material used in this investigation is an industrial aluminum alloy (AA6016 in T4 condition), in the form of rolled sheets of 1 mm thickness. The aluminum was obtained from Novelis (Sierre, Switzerland). It was annealed during manufacturing and not skin passed. The microstructure of the alloy in the as-received state is recrystallized. The composition of the material according to DIN EN 573-3:2007 [36] is given in Table 2. Young’s modulus *E* is determined as 68 GPa with Poisson’s ratio *ν* = 0.33.

**Table 2:** Chemical composition (mass %) of AA6016 according to DIN EN 573-3:2007 [36].

Alloying Component	Si	Fe	Cu	Mn	Mg	Cr	Zn	Al
weight percentage	1–1.5	0.5	0.2	0.2	0.25–0.6	0.1	0.2	balance

### 2.2. Material Testing

**Figure 1:**
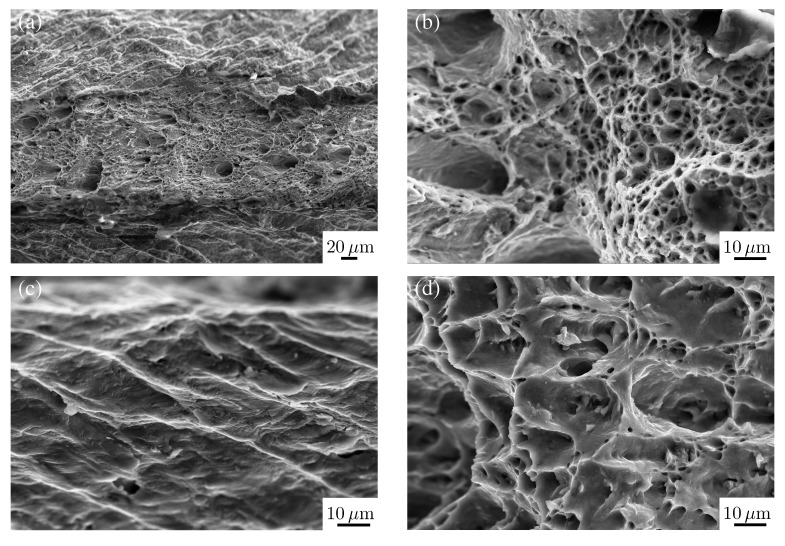
SEM fractograph of the top (**a**,**c**) and center (**b**,**d**) of the fracture surfaces of DC06 (**a**,**b**) and AA6016 (**c**,**d**) in uniaxial tension.

Both materials were subjected to uniaxial tension, plane strain (ps) tension and simple shear loading. Simple shear tests were performed with the shear direction parallel and orthogonal to the rolling direction, where the rolling direction is parallel to the tension direction. The uniaxial tensile tests have been carried out at the Institute of Materials Science, Leibniz Universität Hannover, Germany. The remaining tests were performed with a biaxial testing device (see [37] (Figure 1) for a schematic image of the device) at the Institute of Engineering Technology at Twente University, Netherlands, which is capable of loading a sheet metal specimen in simple shear and in plane strain tension. The biaxial testing device consists of a uniaxial frame, as well as a sub-frame, which is mounted between the cross bars. The sub-frame accommodates an actuator for simple shear deformation. The possibility of buckling during simple shear was minimized by selecting an appropriate ratio of the height *b* = 3 mm in the deformation region to the sample thickness *t* = 1 mm. Homogeneous deformation was achieved in the measurement area due to a large ratio of width to height *c/b* = 15. The final shape of the specimen was obtained by wire eroding in order to minimize the influence of residual stresses at the edges. For further details of the experimental setup, the reader is referred to van Riel and van den Boogaard [37].

**Figure 2:**
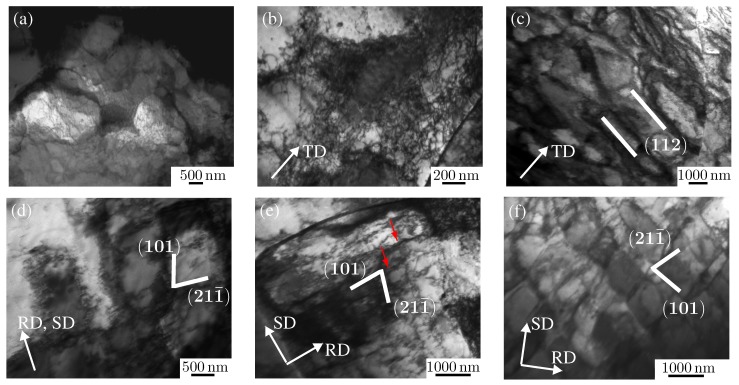
TEM images of dislocation structures of DC06: (**a**) as-received material; after plane strain tension up to 10% (**b**) and up to 20% (**c**) for tension in the rolling direction (RD); after 10% (**d**), 20% (**e**) and 30% (**f**) simple shear loading where shear direction (SD) is orthogonal to the RD. The red arrows in (**e**) indicate a family of dislocation walls parallel to the longitudinal direction of the grain center with a distance of about 50 nm (which is significantly smaller than the distance of 100–200 nm between single dislocation walls of other families).

**Figure 3:**
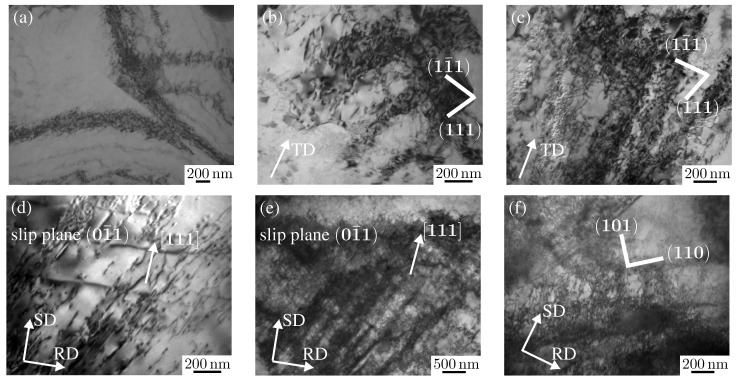
TEM images of dislocation structures of AA6016: (**a**) as-received material; after plane strain tension up to 10% (**b**) and up to 20% (**c**) for tension in the RD; after 10% (**d**), 20% (**e**) and 30% (**f**) simple shear loading, where the SD is orthogonal to the RD.

The tests were performed at constant nominal strain rates of 10^*−*3^ s^*−*1^. The stress-strain responses for DC06 were previously reported in [4] (Figure 2) and the results for monotonic shear of AA6016-T4 in [38] (Figure 3); both showing a typical monotonic increasing stress-strain behavior. The initial yield strengths *σ*_Y_ (at *ε* = 0.002) are identified as *σ*_Y, DC06_ = 145 MPa and *σ*_Y, AA6016_ = 117 MPa. The ultimate tensile strengths *σ*_m_ are determined as *σ*_m, DC06_ = 385 MPa and *σ*_m, AA6016_ = 239 MPa. For completeness, the deformation states and the corresponding normal or shear stress values, at which transmission electron microscopy samples are taken, are given in Table 3.

The microstructure of the samples after fracture under uniaxial tensile loading was investigated on a scanning electron microscope, Zeiss Leo 1455 VP SEM. In order to obtain TEM foils, flat disks with a diameter of 3 mm and a thickness of approximately 500 *µ*m were cut from the center of the deformation zone by wire eroding. These disks were mechanically thinned and polished from both sides to a thickness of 100 *µ*m. The TEM-foils were electropolished with an electrolyte consisting of 120 mL 40% perchloric acid, 440 mL butoxyethanol and 440 mL 100% acetic acid in a Struers Tenupol Electropolisher 5. The microstructural investigations were performed on a JEOL JEM2010 Transmission Electron Microscope with a 200 kV electron gun.

**Table 3:** Relative deformation states of the TEM-samples in (true) stress-strain space (normal strain *ε* to normal Cauchy stress *σ*, resp. shear strain *γ* to shear Cauchy stress *τ* ). ps, plane strain.

Material	Loading Case	Strain *ε* or *γ* (*−*)	Stress *σ* or *τ* (MPa)
DC06	(ps) tension	0.1	392
		0.2	449
	shear	0.1	136
		0.2	166
		0.3	184
AA6016	(ps) tension	0.1	255
		0.2	287
	shear	0.1	130
		0.2	153
		0.3	166

## 3. Experimental Analysis of Microstructural Properties

### 3.1. Fracture Surfaces

The fracture surfaces indicate the nature of the plastic deformation in metallic materials. After the plastic deformation of the aluminum and steel samples up to the point of fracture under uniaxial tension, the features of the topology of the fracture surfaces were analyzed in detail. The corresponding fracture strains are *ε*_fr, DC06_ = 0.554 and *ε*_fr, AA6016_ = 0.283.

The analysis was carried out in three distinct parts (top, center and bottom) of the fracture surface. Figure 1 shows the resulting fractographs at the top and center for DC06 and AA6016. The fractographs of the bottom look similar to the one at the top and are skipped for the sake of brevity. The fractographs of the top and bottom show areas exhibiting facets of cleavage in the steel, as well as in the aluminum alloy (Figure 1a,c). Inclusions are clearly seen in the dimples on the fracture surface. In the case of alloys with the same lattice structure, the difference in the fracture toughness is manifested in the size of the dimples on the fracture surface, even if the lattice structure is changed [39]. Basically, all details of the fracture surface with a strongly curved pattern are cleavage features. Very fine bands are recognizable among the cleavage features exhibiting similarities to patterns observed for material fatigue under cyclic loading (fatigue striations).

The fracture surface’s center shows a ductile fracture dominated by dimples due to microvoid coalescence. These dimples are of an equiaxed shape with a size of approximately 5 *µ*m − 20 *µ*m. The main factor accelerating the generation of dimples due to microvoid coalescence is the large preference of the material to from dimples rather than a different type of fracture. The fracture mechanism is further affected by external factors, such as stress conditions, as well as the strain rate. The pores emerge and grow at inclusions, which are clearly seen in the center of the fracture surface (Figure 1c,d). Additionally, the fracture surface of the steel sample DC06 exhibits deeper dimples in the vicinity of small dimples. Taylor [40] argues that these dimples induce local ductile fracture. In our aluminum samples, smaller parts of the second phase with a size of about 100 nm are observed within the dimples. The main precipitate in 6000 series Al-Mg-Si alloy systems are of type Mg_2_Si; see e.g., [41,42]. Thus, for both materials, a mixed type of fracture is observed. Consequently, some regions in the fracture zone exhibiting ductile fracture originate in the coalescence of micro-voids, as well as separated grain facets, resulting in brittle fracture [40].

Although steel DC06 and aluminum AA6016 show certain differences as they feature different grain sizes and mechanical properties, the structures at the fracture surfaces, however, are similar. From our observation, we conclude that fracture behavior is similar in both materials, indicating that the underlying processes are comparable, as well. Next, the dislocation structures of both materials are investigated under different monotonic loading conditions to clarify the similarities and differences in the dislocation structure.

### 3.2. Microstructural Investigation

In the description of the dislocation microstructure, the following terms are used for microstructural elements: (i) cells are defined as regions of low dislocation density surrounded by lines of higher dislocation density; within the cells, the dislocations are homogeneously distributed; usually neighboring cells have misorientations from 0.2° to 0.8°; (ii) cell walls are regions of higher dislocation density surrounding the cells; the misorientation from the cell interior to the cell wall can be as large as 1°–1.5°; and (iii) cell-block boundaries are dense dislocation walls, which surround an area of dislocation cells; they are parallelepiped in shape and confine planar persistent dislocation structures.

#### 3.2.1. IF-Steel DC06: TEM Image and Analysis

Figure 2a shows a TEM micrograph of the as-received material DC06. The initial texture is a *γ*-fiber texture with 〈111〉 direction oriented parallel to the sheet normal direction, which is characteristic for cold-rolled IF-steels [4]. As stated by Nesterova *et al*. [7], the orientation of the grains along the *γ*-fiber influences the dislocation distribution at shear strains up to 30%. The average grain size is determined as 20 *µ*m, and the grain sizes range from 5 *µ*m–60 *µ*m, where the maximal and minimal grain size is only found rarely. The average deviation from the average grain size is very small. All specimens used in this work were manufactured from the same batch of material with average *r*-values (*i.e*., anisotropy parameter): *r*_0°_= 2.31, *r*_45°_ = 1.95, *r*_90°_ = 2.77.

In uniaxial tension, the material tends to form cell-block boundaries initially predominantly associated with {110} glide systems. As deformation continues, cell-block boundaries belonging to {112} systems are observed. After 15% strain, cell-block boundaries oriented on {123} planes appear, as well. In general, the cell-block boundaries are observed after 5% strain, but they are more pronounced at larger strains (e.g., 20%). At strains between 5% and 8%, cells and planar persistent structures coexist. Additionally, between 5% and 12% tensile strain, dislocations tangles are observed, as well. Our analysis indicates that these appear in certain parts of the sample in this strain range, where other tangles seem to disappear simultaneously. After 12% tensile strain, nearly all dislocation tangles are incorporated into cell walls. With increasing deformation, the misorientation angle between cell interior and cell-block boundaries increases. Finally, this results in sharp boundaries referred to as blade-like plates on a larger scale (*cf*. Rybin [43]). The rolling texture increased with tensile loading, as reported in [4], where a strengthening of the *γ*_2_-component (see [7] for terminology) is found to accumulate around *ϕ*_2_ = {0°, 60°}.

Consistent with observations of Bouvier *et al*. [34], during plane strain tension, the degree of irregularity of the dislocation microstructure increases compared to uniaxial tensile loading (see Figure 2b,c). Cell block boundaries start to form analogously. Under plane strain tension, a second group of cell-block boundaries oriented in another direction of the family 〈111〉 is observed more often. The cell-block boundaries exhibit more curvature and are more diffuse than in uniaxial tension. The electron-microscopical investigations show that up to a tensile strain of 10%, grain sizes of 10 *µ*m *−* 12 *µ*m are observed. In deformed grains, cell walls are formed by kinked arrangements of dislocations. In these regions, dislocations rearranging into sub-grain-like boundaries are observed. They are referred to as dense dislocation walls. In elongated grains with a high aspect ratio, the grains are fragmented into subgrains of about 1 *µ*m in width. The boundaries of these subgrains appear as dislocation walls and networks, which sometimes form subgrain boundaries with a fringe contrast. The dense dislocation walls and networks are inclined 15°–20° to the macroscopic tensile axis. As reported by Clausmeyer *et al*. [4], at the same normal strain, the dislocation structure under uniaxial tension consists of twice as much cells as under plane strain tension. With increasing deformation up to 20% strain, the cell size decreases and the dislocation density in areas of tangles and accumulations increases. This is accompanied by a tendency to arrange parallel subboundaries. The grain size also decreases to 6 *µ*m–8 *µ*m. Dense dislocation walls are parallel to traces of {110} planes, which is the rolling plane (trace of plane). The rolling texture increases under plane strain tension with a strengthening of the *γ*_2_-component as under uniaxial tension.

After simple shear deformation of 10% strain, grain sizes of up to 10 *µ*m are observed analogous to tensile loading. Figure 2d shows a typical pattern for the dislocation structure after 10% shear strain. The development of two families of dense dislocation walls within a single grain are observed (trace of plane). These dislocation walls incline 5°–8°in the longitudinal plane toward the shear direction. An important requirement for the creation of a certain texture or microstructure is the interaction of several slip systems [44]. With shear deformation up to 20% strain, the grain size decreases slightly (e.g., to 9.4 *µ*m), while the dislocation density in the grains, as well as on the grain boundaries increases. Dense dislocation walls are inclined 10°–25° with respect to the shear direction. Between 20% and 30% shear strain, grain sizes up to 10 *µ*m are measured and the intensity of dislocation networks increases. Additionally, grains with single dislocations are found. The forming cell blocks show a rectangular shape (*i.e*., aspect ratio close to one) compared to the quadrilateral shape after tensile loading. Under shear around 50%, more cells have developed compared to tensile loading at the equivalent amount of strain. The formation of micro shear bands starts at shear strains of about 30%. They enclose angles of 35°–45° with the plane of deformation {110}. Noteworthy is the fact that their formation has no relation with pre-existing dislocation cells. Figure 2e,f show the dislocation structure of a micro-band in the specimen. At 20% strain, the nucleation of a micro-band can be anticipated, where the full one is created at larger strain values. The boundaries of the band are dense dislocation walls and are approximately parallel to {110} planes. Each band consists of two families of dislocation walls (oriented 35° to 〈110〉. One family parallel to the longitudinal direction of the grain (see Figure 2e) has a distance of about 50 nm between single dislocation walls compared to the family that is significantly smaller than the distance of 100 nm–200 nm between single dislocation walls. The initiation of micro-bands often starts at large precipitates or grain boundaries. Precipitates and grain boundaries serve as dislocation sources contributing to a higher dislocation density on a single slip system. This might result in the formation of dense dislocation walls and, finally, to the appearance of micro-bands. Micro-bands, not initiated by large precipitates or grain boundaries, can be found for small carbide particles of about 5 nm–50 nm. Figure 2f shows two families of micro-bands within a grain at 30% shear deformation. The micro-bands are 100 nm–400 nm thick. The area of micro-bands increases and the morphology becomes similar to an arrangement of blade-like plates of dense dislocation walls. In Figure 2f, the micro-band walls are located parallel to {101} planes. Localization of deformation in narrow bands (deformation bands) starts when parts of a single crystal start to reorient due to several reasons, such as anisotropy, texture and strain rate. This process is called orientation split-up. Two families of cell-block boundaries are predominant, either parallel or orthogonal to the shearing direction. At large amounts of shear, such as 50%, in some grains, both families are found at the same time. For large amounts of deformation, misorientations from 0.8° up to 5° are observed within the cell-block boundaries, with most misorientations being smaller than 3°.

#### 3.2.2. Aluminum Alloy AA6016-T4: TEM-Image and Analysis

An analogous investigation was performed on the fcc aluminum alloy AA6016-T4, and the results are correlated to the previous one from DC06. Figure 3a shows a TEM micrograph of the as-received material AA6016-T4, which exhibits a grain size of approximately 20 *µ*m–25 *µ*m and average *r*-value *r*_0°_ = 0.63, *r*_45°_ = 0.41, *r*_90°_ = 0.77 [38]. The electron-microscopical investigation of the alloy after plane strain tension after 10% deformation shows that dislocation walls and dislocation nets are observed in the microstructure. The dislocation walls are inclined about ±10° to the loading direction. In addition, the number of grains exhibiting a cell structure is significantly higher than in DC06. With increasing deformation up to 20%, the dislocation density is increased (see Figure 3b). Most dislocation walls are now inclined about ±40° to the loading axis. The formation of dislocation structures is intensified. Often, two families of dislocation walls are found in the microstructure, namely on {111} in two directions of the family 〈110〉. These walls are parallel to traces of {111} slip planes and not to the tensile direction. Changes in the degree of deformation of the aluminum alloy sample result in high dislocation densities, dislocation clusters redistribution inside the cell-structure and cell boundaries similar to those observed in DC06.

After a plastic deformation of 10% shear, the grains feature an approximate size of 1 *µ*m and the dislocation structure is identified as a dislocation net (see Figure 3d). In addition to the formation of dislocation walls and dislocation nets, single dislocations are observed. As in the case of plane strain tension, dislocation nets in the longitudinal planes are inclined about ±10° to the rolling direction. With increasing shear deformation up to 20%, grains are of size of about 6 *µ*m–8 *µ*m, which probably increased due to recrystallization effects. Furthermore, the dislocation density increases. The formation of dislocation walls and dislocation nets are intensified. Two families of nets are seen in Figure 3e; however, in most of the cases investigated, only one family is observed. This family of net is formed with an inclination angle of 35° with respect to the macroscopic shear direction. However, in some grains, micro-bands begin to form (see Figure 3f) where two families of micro-bands are observed within a grain, as well. The thickness of the micro-bands is identified as about 50 nm. The micro-band walls are parallel to {110} planes. The trace of the second family of micro-bands are parallel to the {101} planes.

## 4. Discussion

The characteristic features of the dislocation structure and its dynamic evolution under the two loading conditions plane strain tension and simple shear are summarized in Table 4 for aluminum alloy AA6016-T4 and IF-steel DC06. In the uniaxial tension tests, the dislocation structures develop more intensively and quickly compared to the plane strain test. In DC06, dislocation cells start to develop in multiple grains with a favorable Schmid factor at 5% strain in uniaxial tension test, whereas in plane strain tension, this is only seen at approximately 10% strain. In the case of AA6016, the same, but less pronounced behavior is observed.

**Table 4:** Characteristics of dislocation structures under plane strain deformation in aluminum alloy AA6016-T4 and steel DC06.

Loading Conditions	Strain Level	Characteristics of Dislocation Structure	
		Aluminum Alloy AA6016-T4	Steel DC06
plane strain tension	0	single dislocations, accumulations of dislocations	single dislocations, cell structure
	0.1	single dislocations, start of formation of dislocation walls	cell structure and start of formation of dislocation walls
	0.2	formation of dislocation cells parallel to two directions	cell structure, interaction between dislocation walls, micro-shear bands
Shear	0.1	single dislocations, start of formation of dislocation walls parallel to two directions, cell structures	single dislocations, dislocation pile-up
	0.2	formation of dislocation cells, dislocation walls	dislocation pile-up, cell structures
	0.3	on basis of the dislocation walls form micro-shear bands	dislocation pile up, cell structure and start of formation of dislocation walls

Plane strain tension in DC06 leads to a less intensive deformation at the micro-level compared to uniaxial tensile loading. Nevertheless, at strains higher than 10%, a detailed measurement of dislocation densities is not possible due to the fact that dislocation walls and cells with high dislocation densities have already developed. The cells show fewer dislocations in the interior and are formed in a polygonal shape, with most being rectangular (cell blocks). These cells are larger than the created fragments in uniaxial tension at the same strain level. The intensity of plastic deformation is larger under tension compared to shear, because under tension, more pronounced and intense dislocation structures appear. Already at low strains (*approx*. 10%), the formation of dislocation walls is observed. The number of cells and the aspect ratio *a/b* of the cells are compared in Table 5 for DC06 and AA6016-T4 under plane strain tension and shear at various deformation states. Here, *a* denotes the size in or close to the direction of loading and *b* denotes the size perpendicular to that. Under simple shear, the characteristic dislocation cell shape is close to a quadratic shape.

In AA6016-T4, the formation of dislocation structures is occurring at a higher intensity in the plane strain tension test compared to the one in shear. Already at low strains, dislocation walls of high dislocations (approximately 10^14^ m*/*m^3^ to 3 × 10^14^ m*/*m^3^; see [45] and [46] for details about dislocation density calculations from TEM images) are observed in plane strain tension. Under shear loading, the dislocations form quadratically-shaped dislocation walls at strains up to 20%, consisting partially of single dislocations. These cell walls are smaller (250 nm–500 nm for plane strain tension, 150 nm–300 nm for shear) and have a lower dislocation density (approximately 2 × 10^13^ m*/*m^3^ to 5 × 10^13^ m*/*m^3^) compared to the ones under plane strain tension. A further strain increase leads to the formation of micro-shear bands, which are formed on the basis of the previously-formed dislocation cell walls. The cell shapes under plane strain tension are rectangular with an aspect ratio of 1:2. Under simple shear, the cells formed first have an aspect ratio of 1:3, and with increasing deformation, the cells become quadratic.

**Table 5:** Microstructural properties of the dislocation structures under plane strain tension, resp. shear loading, in aluminum AA6016-T4 and steel DC06 at various strain levels.

Loading Conditions	Strain	Number of Cells Per Grain		Aspect Ratio *a/b* of Cells		Orientation of Cells to Loading Direction	
		AA6016-T4	DC06	AA6016-T4	DC06	AA6016-T4	DC06
plane strain tension	0.1	10 to 20	2 to 6	1:2 to 1:1	1:2 to 1:3	10°	10°–15°
	0.2	25 to 30	6 to 8	1:2	1:1 to 1:4	35°–40°	15°–20°
shear	0.1	20	-	1:3 to 1:2	1:1, rarely 1:2	3°–10°	5°–8°
	0.2	30 to 40	5	1:2	1:1	35°–40°	10°–25°
	0.3	50 to 60	15	1:1	1:1, rarely 1:2	35°–40°	30°–35°

**Figure 4:**
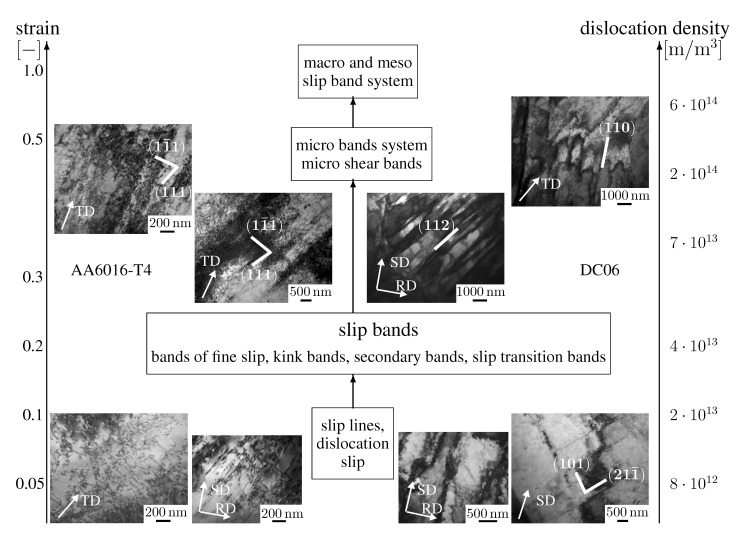
Classification of microstructural elements in aluminum alloy AA6016-T4 and steel DC06 observed at different levels of deformation. These are correlated with the observed dislocation densities.

Comparing the results of the two materials with different lattice structures (bcc and fcc), it is noted that the deformation in DC06 is more intense than in AA6016-T4. A classification of the microstructural features of both materials is suggested in Figure 4. The focus is on a qualitative description of the dislocation structure without an exact quantification of the dislocation density. In general, differences in the intensity of the formation of dislocation structures are observed in plane strain and shear loading. The investigation of the dislocation densities in this work is based on two to three measurements at each strain level. This corresponds to the analysis of ten to twelve pictures of the dislocation structure tilting differently from the specimen holder.

By comparing the intensity in plane strain tension of the formation of dislocation structures between both materials, the following observations can be made:

- In the as-received material, as well as at low strain levels (0%–5%), single dislocations and accumulations of dislocations are observed in aluminum, whereas in steel, already dislocation cells are starting to form.
- An increase of the strain level up to 10% leads to the formation of dislocation walls in both materials.
- A further increase in strain leads to differences in the dislocation structure evolution: in aluminum, an orientation/direction-dependent formation of dislocation cells is occurring; in steel, a strong interaction between the dislocation walls and the formation of micro-shear bands are observed.

The formation of the dislocation structures under shear in aluminum and steel show very different tendencies compared to the ones under plane strain:

- In aluminum, the formation of dislocation cells starts earlier than in steel, even for strain levels smaller than 0.1.
- An evolution of the dislocation structure is not based on the intensive formation of dislocation cells and dislocation walls. Dislocation walls in combination with micro-shear bands in aluminum and in steel form due to the formation of strongly-branched dislocation structures.

The comparison of the hardening in both materials shows that the hardening behavior is similar, although differences are seen in the intensity of the microstructural processes. The dislocation structures in DC06 and AA6016-T4 show certain differences; however, the dynamics in the hardening behavior is similar. This indicates that the principles in the plastic deformation are rather defined by the form and distribution of dislocation structures than by the glide mechanism alone. Both materials exhibit a monotonic hardening behavior, where hardening saturates with increasing amounts of strain.

## 5. Summary and Outlook

On the basis of plane strain tension and simple shear tests, the evolution of the grain and dislocation microstructures have been investigated for two materials with different lattice structures, namely an interstitial free steel (DC06) and a 6000 series aluminum alloy (AA6016-T4). Within this work, the microstructural changes and features at different strain states were characterized and compared for both materials to provide information on the active mechanism at the microscale governing the macroscopic response. The observations suggest that the plastic deformation mechanisms are rather defined by the form and distribution of dislocation structures rather than by the glide mechanism alone. Overall, the characterization of dislocation structure revealed that the deformation in DC06 is more intense than in AA6016-T4. Different tendencies in the dislocation formation were obtained under plane strain tension compared with shear loading for both materials, which has been discussed in detail. Further, it was found that the fracture behavior of DC06 and AA6016 is similar under uniaxial tensile loading, indicating that the processes are similar, as well.

As a next step, these results can be used to motivate and validate new micromechanical-based numerical models. A thoughtful material model for the macroscopic response requires the modeling of the microstructural mechanisms resulting in dislocation patterns. Therefore, a detailed investigation for different loading histories, as presented in this work, provides a good basis. Only such detailed local analyses allow for the determination of differences in the behavior of materials and provide conclusive results regarding the responsible physical mechanisms.
